# Intelligent Priority-Aware Spectrum Access in 5G Vehicular IoT: A Reinforcement Learning Approach

**DOI:** 10.3390/s25154554

**Published:** 2025-07-23

**Authors:** Adeel Iqbal, Tahir Khurshaid, Yazdan Ahmad Qadri

**Affiliations:** 1 School of Computer Science and Engineering, Yeungnam University, Gyeongsan-si 38541, Republic of Korea; 2 Department of Electrical Engineering, Yeungnam University, Gyeongsan-si 38541, Republic of Korea; tahir@ynu.ac.kr

**Keywords:** 5G, Internet of Things, priority-aware spectrum management, reinforcement learning, spectrum access, resource allocation

## Abstract

Efficient and intelligent spectrum access is crucial for meeting the diverse Quality of Service (QoS) demands of Vehicular Internet of Things (V-IoT) systems in next-generation cellular networks. This work proposes a novel reinforcement learning (RL)-based priority-aware spectrum management (RL-PASM) framework, a centralized self-learning priority-aware spectrum management framework operating through Roadside Units (RSUs). RL-PASM dynamically allocates spectrum resources across three traffic classes: high-priority (HP), low-priority (LP), and best-effort (BE), utilizing reinforcement learning (RL). This work compares four RL algorithms: Q-Learning, Double Q-Learning, Deep Q-Network (DQN), and Actor-Critic (AC) methods. The environment is modeled as a discrete-time Markov Decision Process (MDP), and a context-sensitive reward function guides fairness-preserving decisions for access, preemption, coexistence, and hand-off. Extensive simulations conducted under realistic vehicular load conditions evaluate the performance across key metrics, including throughput, delay, energy efficiency, fairness, blocking, and interruption probability. Unlike prior approaches, RL-PASM introduces a unified multi-objective reward formulation and centralized RSU-based control to support adaptive priority-aware access for dynamic vehicular environments. Simulation results confirm that RL-PASM balances throughput, latency, fairness, and energy efficiency, demonstrating its suitability for scalable and resource-constrained deployments. The results also demonstrate that DQN achieves the highest average throughput, followed by vanilla QL. DQL and AC maintain fairness at high levels and low average interruption probability. QL demonstrates the lowest average delay and the highest energy efficiency, making it a suitable candidate for edge-constrained vehicular deployments. Selecting the appropriate RL method, RL-PASM offers a robust and adaptable solution for scalable, intelligent, and priority-aware spectrum access in vehicular communication infrastructures.

## 1. Introduction

Sixth-generation (6G) wireless networks are aimed at revolutionizing communication by providing ultra-reliable low-latency communication (URLLC), massive connectivity, and cognitive decision-making for numerous emerging applications such as autonomous systems, healthcare, and intelligent mobility [1]. Innovations in intelligent reflecting surfaces (RIS), massive multiple-input multiple-output (MIMO) architectures, integrated sensing and communication (ISAC), and AI-driven automation are expected to support highly dynamic and mobile communication environments [2]. In 6G, the Internet of Things (IoT) is expected to enable distributed sensing, actuation, and control in numerous environments, ranging from industrial automation and smart cities to healthcare and vehicular networks.

With the exponential growth in the deployment of IoT devices, IoT ecosystems are facing increasing challenges like heterogeneous traffic types, spectrum scarcity, and the demand for low-latency energy-efficient communication. These are especially critical in resource-constrained environments, where access to the spectrum needs to be adaptive in order to guarantee Quality of Service (QoS). Scalable over-the-air aggregation and low-complexity state estimation for massive-scale IoT installations have recently been explored to support efficient coordination in massive IoT scenarios [3], highlighting the importance of intelligent resource management techniques.

Vehicular Internet of Things (V-IoT) refers to the integration of vehicles into the IoT ecosystem, enabling real-time communication, sensing, and data exchange among vehicles, infrastructure, and cloud services to support intelligent transportation systems. In V-IoT, high mobility, safety-critical communications, and dynamic traffic conditions worsen the spectrum access problems. Vehicles become consumers and information relays for real-time information to a large degree, requiring smart, low-latency, and fair access to spectrum resources. Thus, V-IoT systems must use adaptive and context-aware technologies that can effectively operate under time-varying channel conditions and varying traffic loads.

V-IoT forms the backbone of modern intelligent transportation systems and smart city infrastructures, supporting critical services such as autonomous driving, real-time collision avoidance, traffic optimization, infotainment, and predictive diagnostics [4,5]. With projected traffic volumes in V-IoT networks surpassing several exabytes per month and a significant share of applications requiring latency below ten milliseconds, there is an urgent need for adaptive and intelligent spectrum management mechanisms [6,7].

Traditional spectrum allocation strategies, such as fixed-assignment and reservation-based models, as well as cognitive radio (CR) protocols, are ill-equipped to handle the dynamic and heterogeneous requirements of V-IoT networks. As vehicular density increases and communication patterns become more bursty and context-sensitive, the conventional approaches struggle to maintain Quality of Service (QoS) across the board, including in terms of reliability, latency, and throughput. The hybrid underlay–interweave access models proposed for CRs offer improved flexibility by incorporating context-aware rules for spectrum reuse and sharing [8,9,10]. To address similar dynamic challenges in hybrid CRNs, recent works have leveraged cooperative and deep reinforcement learning, such as Actor–Critic [11], DQN [12] and Deep Deterministic Policy Gradient (DDPG) [13], to support distributed decision-making, power control, and adaptive access in complex and large-scale environments, highlighting the adaptability and robustness of RL in managing complex spectrum environments. Priority-aware spectrum management (PASM) techniques extend this paradigm by introducing service differentiation based on traffic urgency, granting privileged access to high-priority applications while attempting to sustain fairness and efficiency [14,15].

Despite their advantages, conventional PASM techniques typically rely on manually designed and immutable policies and rules, and thus limit them from embracing real-time environmental variations and random traffic demands [16]. To overcome such limitations, artificial intelligence (AI) and reinforcement learning (RL) in particular have been viewed as an exciting alternative. RL is capable of enabling autonomous agents to learn optimal access policies through exploration of the environment and successive refinement of decisions in response to reward feedback. As compared to rule-based models, RL agents can dynamically adapt themselves based on changing traffic, spectrum availability, and service needs without prior knowledge of them or having pre-defined models of network behavior.

In reinforcement learning, the agents learn to choose the best possible actions in an environment by exploring it and receiving feedback in the form of rewards. This decision-making process is often captured by a Markov Decision Process (MDP), a mathematical abstraction of modeling the environment as a state-action pair, transition probabilities, and rewards [17]. MDPs perform extremely well for dynamic spectrum access challenges where the agent has to make a series of decisions under uncertainty, e.g., varying channel availability, diverse traffic demand, and varying QoS requirements. With MDPs, RL agents are able to create context-based spectrum-access rules that adapt to fluctuating vehicular traffic and channel states.

Recent works have demonstrated the viability of RL-based spectrum access in vehicular networks, with algorithms such as Q-Learning, Deep Q-Network (DQN), and Actor–Critic achieving notable improvements in throughput, delay, fairness, and reliability [18,19,20,21]. Extensions, such as multi-agent RL and federated RL, further address challenges related to scalability and data privacy in distributed vehicular deployments [22,23]. Moreover, real-world testbed implementations have begun validating the feasibility of RL-based spectrum management under practical constraints [24,25]. Nonetheless, existing studies often focus on a single RL paradigm or QoS metric, lacking a holistic framework that simultaneously accounts for throughput maximization, delay minimization, fairness preservation, and energy efficiency in a unified setting. Furthermore, the integration of multiple RL algorithms within a common architecture for comparative analysis under realistic vehicular load conditions remains unexplored.

To bridge this gap in the literature, we propose a centralized PASM framework based on RL (RL-PASM). The RL-PASM framework operates at the Roadside Unit (RSU), which serves as a centralized agent that learns and enforces spectrum access policies in real-time. The model supports three distinct traffic classes, high-priority (HP), low-priority (LP), and best-effort (BE), and evaluates the performance using four RL agents: Q-Learning (QL), Double Q-Learning (DQL), DQN, and AC. Spectrum access is formulated as a finite-state Markov Decision Process (MDP), with a multi-objective reward function designed to optimize performance across multiple QoS dimensions. The key contributions of this work are summarized as follows:

1. We introduce a centralized, RL-driven PASM framework tailored for 5G- and 6G-enabled V-IoT environments, incorporating multi-priority class support and RSU-based spectrum coordination.
2. We design a novel multi-objective reward function that jointly considers throughput, latency, fairness, and energy efficiency to guide the agent’s decision-making process.
3. We implement and benchmark four RL strategies, QL, DQL, DQN, and AC, to simulate spectrum access in realistic vehicular traffic dynamics and network parameters. Comprehensive evaluations are performed across key performance metrics, including throughput, delay, blocking probability, fairness index, energy efficiency, interruption rate, channel utilization, and convergence behavior.

The rest of the manuscript is structured as follows: Section 2 reviews the relevant literature on spectrum access and RL in V-IoT. Section 3 presents the system model and MDP formulation. Section 4 introduces the proposed RL-PASM framework and associated learning algorithms. Section 5 details the simulation environment and performance metrics. Section 6 discusses the experimental results. Finally, Section 7 concludes the paper and outlines future research directions.

## 2. State-of-the-Art Review

RL has emerged as a promising solution for enabling adaptive and priority-aware spectrum access in V-IoT networks. These networks are characterized by dynamic mobility, heterogeneous service demands, and varying priority levels, which make conventional rule-based spectrum access schemes insufficient. Classical QL, known for its simplicity and model-free nature, has been effectively applied to vehicular scenarios where devices autonomously learn optimal access strategies through trial and error. Ref. [18] introduces QLOCA, a QL–based algorithm for priority-aware channel selection, and demonstrates significant gains in throughput and latency reduction over static baselines under variable vehicular traffic conditions.

To overcome the limitations of tabular Q-Learning in high-dimensional environments, DQN, a neural network-based RL algorithm, is used to approximate action-value functions. Ref. [26] explores DQN in cellular-V2X networks, achieving enhanced spectral efficiency by enabling vehicles to switch intelligently between access modes under varying traffic and interference conditions. Ref. [20] extends this work by proposing a multi-reward DQN for cognitive vehicular networks. Their approach jointly optimizes throughput, delay, and fairness, resulting in faster convergence and improved access reliability. Multi-agent RL (MARL) has also gained traction in decentralized V-IoT environments. Ref. [19] adopts a centralized training and decentralized execution (CTDE) framework using QL, which reduces access collisions and enhances fairness as measured by Jain’s Fairness Index. Advanced approaches such as Dueling Double DQN integrate a dueling architecture and double estimators to optimize sub-channel allocation and power control jointly. Their method demonstrated both faster convergence and improved capacity under realistic vehicular communication loads [21].

Recent work has also advanced RL-based dynamic spectrum access (DSA) to more sophisticated and heterogeneous IoT environments. Ref. [11] proposes CoMARL-DSA, a cooperative multi-agent reinforcement learning paradigm suited for cognitive radio networks. Their system, based on a decentralized partially observable Markov Decision Process (Dec-POMDP), employs a centralized training and distributed execution framework through Deep Recurrent Q-Networks (DRQN), reducing collision rates to the bare minimum while maximizing overall system throughput in decentralized multi-user environments. Similarly, ref. [12] develops a unified Double DQN-based framework for D2D-assisted IoT networks that supports orthogonal and non-orthogonal spectrum access in both uplink and downlink transmissions. The framework not only optimizes sum throughput, but also integrates fairness constraints through a novel reformulation of the Q-function. Concurrently, ref. [13] explores spectrum efficiency and energy efficiency in RF-powered ambient backscatter-assisted hybrid underlay CRNs, proposing an adjusted DDPG algorithm as well as a hybrid convex optimization framework. These works collectively suggest the onset of advanced deep RL techniques tailored to spectrum reuse, fairness-aware access, and energy-constrained communication environments.

Addressing fairness explicitly, ref. [27] proposes a reward-adjusted Q-Learning approach for edge-IoT networks operating under underlay and overlay cognitive radio modes. Their scheme achieved a balanced trade-off between rate and utility across devices with differing priorities. Beyond simulation-based investigations, real-world implementations and analytical modeling have validated the feasibility of RL in V-IoT. Ref. [24] implements a QL–driven spectrum hand-off mechanism using USRP hardware, demonstrating real-time decision-making capabilities and tangible improvements in hand-off success rates. Complementing these empirical results, ref. [25] provides theoretical insights by modeling primary user activity in vehicular ad hoc networks (VANETs) to guide RL-based access policies. Their work highlights how accurate modeling can significantly improve spectrum reliability and utilization. The studies above collectively demonstrate the versatility of RL in achieving adaptive, scalable, and priority-sensitive spectrum access. Table 1 contains a summary of the state-of-the-art contributions. These proposals establish the foundation and motivation for the proposed RL-PASM framework, which aims to unify and extend these methods into a centralized, multi-priority access system adapted to 5G-enabled vehicular networks.

## 3. System Model

**Figure 1:**
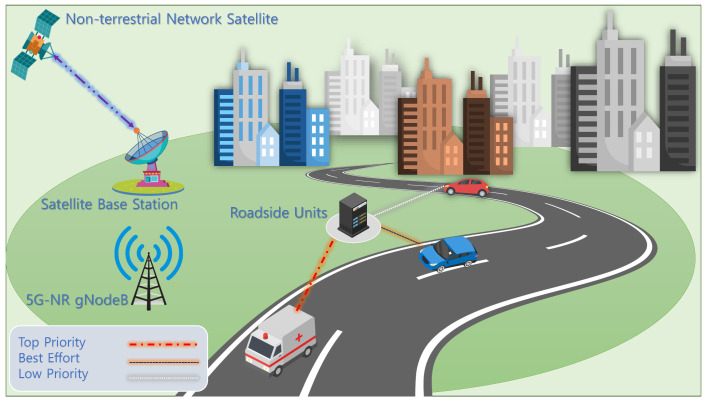
System model of RL-PASM showing RSU-based spectrum allocation for HP (red), LP (blue), and BE (white) traffic classes.

**Figure 2:**
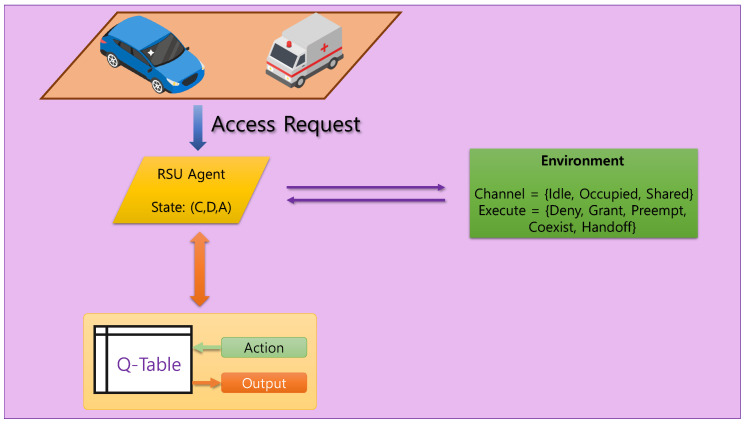
Centralized RL-PASM architecture: The RSU observes the system state (C,D,A) and selects an access policy based on learned priorities for HP, LP, and BE vehicular traffic.

Consider a centralized spectrum management architecture for V-IoT networks, where RSUs act as intelligent decision-making agents responsible for regulating channel access among vehicles, as shown in Figure 1. The V-IoT environment includes autonomous and semi-autonomous vehicles, each categorized into one of three communication classes: HP, LP, and BE. HP traffic corresponds to ultra-low latency safety messages, LP traffic covers time-sensitive non-critical data, while BE traffic involves delay-tolerant applications. At every discrete time step, a vehicle issues a channel access request to the RSU. In the simulation environment, channel access requests follow a stochastic process modeled using a Bernoulli distribution for device activity and a categorical distribution for traffic classes and channel states. This setup reflects the dynamic and heterogeneous nature of V-IoT traffic in time-varying environments anddetermines an appropriate response based on the current channel occupancy and the requesting device’s priority class, as illustrated in Figure 2. This decision process is modeled as a finite MDP, where the environment state is encoded as a tuple (C,D,A).

The environment state is encoded as a triplet (C,D,A), where *C* denotes the channel condition (idle, occupied, or shared), *D* represents the priority class of the requesting vehicle, and *A* denotes the priority class of the currently occupying vehicle, if any. Each of these variables takes values in {0, 1, 2}, corresponding to three distinct levels. To enable compact indexing in tabular reinforcement learning, the state is linearly mapped to an integer using base-3 encoding:

(1) S=9×C+3×D+A

This yields 3×3×3=27 possible unique states, capturing the heterogeneity in both channel conditions and traffic classes. This encoding provides a structured and efficient representation for state-action learning under dynamic vehicular spectrum conditions.

The RSU chooses one of five actions at each time step, corresponding to

1. Deny: Reject the access request regardless of status.
2. Grant: Allow access if the channel is idle.
3. Preempt: Forcefully replace the current user if the requester has a higher priority.
4. Coexist: Permit concurrent access, accepting potential degradation.
5. Handoff: Attempt redirection to a different channel with additional switching cost.

The agent seeks to learn an optimal policy $\pi^{*}:S\to A$ that maximizes the cumulative expected reward:

(2) $$\pi^{*}\left(S\right)=\arg\max_{\pi }E\left[\sum_{t=0}^{\infty }\gamma^{t}R\left(S_{t},a_{t}\right)\right]$$

where the immediate reward is $R(S_{t},a_{t})$, it is determined by the outcome of the selected action at time *t*. γ∈(0, 1) is the discount factor that prioritizes future versus immediate gains, and it weights the future rewards.

This abstract enables the integration of various RL algorithms like QL, DQL, DQN, and AC to enable self-decision-making in dynamic vehicular environments. The expressive but compact MDP definition enables real-time spectrum adaptation with minimal computational complexity, which is suitable for smart V-IoT applications.

## 4. Proposed RL-PASM Framework

To mitigate the conflicts of dynamic and priority-sensitive spectrum access for V-IoT environments, we suggest a centralized RL-PASM framework. In this suggested framework, the RSU is an intelligent decision maker, learning optimum access control policies in real-time to respond to varying vehicular loads, channel conditions, and QoS requirements.

### 4.1. MDP-Based Problem Formulation

The RL-PASM approach formulates the spectrum access problem as a finite MDP with state set S, action set A, reward function R(S,a), and probabilistic transition model conditioned on vehicular motion and spectrum availability. The RSU senses the environment state $S_{t}\in S$ at every discrete time step *t* and selects an action $a_{t}\in A$ according to its current policy.

The agent aims to learn an optimal policy π:S→A that maximizes the long-term discounted cumulative reward described in Equation (2).

### 4.2. State and Action Space

Each state *S* represents a snapshot of the system environment and is defined by a triplet (C, D, A):

- C∈{0, 1}: Channel status.
- D∈{0, 1, 2}: Priority class of the requesting vehicle.
- A∈{0, 1, 2}: Class of the currently occupying vehicle (if any).

The action set consists of

A={Deny,Grant,Preempt,Coexist,Handoff}

These actions determine whether the channel request is denied, granted, forcefully preempted, shared, or handed off to an alternate band.

### 4.3. Reward Function Design

The reward function is crafted to reflect the QoS requirements and operational efficiency across different traffic classes. The reward is tabulated as

(3) $$R\left(S,a\right)=\left\{\begin{array}{cc} +10, & \mathrm{if}\;a=\mathrm{Grant}\wedge C=0\\ +5, & \mathrm{if}\;a=\mathrm{Preempt}\wedge D=\mathrm{HP}\\ +2, & \mathrm{if}\;a=\mathrm{Coexist}\wedge D=\mathrm{BE}\\ +5, & \mathrm{if}\;a=\mathrm{Handoff}\\ -10, & \mathrm{otherwise} \end{array}\right.$$

The fixed reward weights were selected through iterative tuning and were informed by the prior RL-based spectrum access literature [28,29]. They were empirically validated to ensure sufficient reward gradients for effective learning under diverse vehicular traffic scenarios. This reward formulation implicitly encodes a multi-objective optimization strategy, where each action-reward pair reflects a distinct QoS goal: throughput is incentivized through idle channel grants, latency is reduced by prioritizing urgent requests, fairness is addressed via coexistence for BE users, and energy efficiency is encouraged by penalizing unnecessary or costly actions.

The advantage of this multi-objective reward function is that it can capture inherent trade-offs in dynamic Vehicular Spectrum Access. It does not optimize individual metrics like throughput at the expense of others like delay or fairness, and thereby promotes well-balanced, context-sensing decision-making aligned with real-world V-IoT requirements.

The reward structure encourages the following:

1. Granting idle channels to reduce delay and improve throughput;
2. Preempting low-priority users in favor of HP devices to ensure urgent access;
3. Allowing for coexistence to maximize utilization for BE traffic;
4. Supporting hand-off decisions moderately;
5. Penalizing ineffective or resource-wasting actions.

This formulation strikes a balance between adaptability, priority differentiation, and spectrum efficiency.

### 4.4. Learning Algorithms

The four distinct RL algorithms implemented using the RL-PASM framework are Q-Learning, Double Q-Learning, DQN, and AC. These agents have been carefully selected to span a range of design paradigms from lightweight tabular methods to more complex function approximation techniques, so that the efficiency of learning, computation overhead, and deployability onto RSUs with varying resources could be explored in depth.

Q-Learning is an initiation-point tabular algorithm and is more famously recognized due to its efficiency and simplicity in discrete settings. Q-Learning operates by iteratively establishing a Q-table that maps state–action pairs to expected cumulative rewards and implementing a temporal difference learning rule. Due to the fact that it is model-free and uses discrete representations, Q-Learning is computationally inexpensive, with low process and memory demands. This makes it particularly attractive for RSUs with sparse hardware resources and those under hard latency and power constraints. Q-Learning, however, has been shown to be overestimation-biased in action values, especially in stochastic environments.

To address this vulnerability, the framework provides Double Q-Learning, an extension of the standard algorithm that corrects the overestimation bias by decoupling the action selection process from the evaluation process. Specifically, it maintains two separate Q-tables and alternately updates them during learning. When selecting an action from one table, its estimation is taken from the other, reducing the positive bias that can skew learning and cause suboptimal policies. Like Q-Learning, Double Q-Learning is computationally and memory-light, but typically results in more stable learning and improved policy accuracy in partially observable or high-variance environments.

Beyond these tabular methods, the RL-PASM architecture integrates DQN to explore the benefit of value function estimation using neural networks. DQN replaces the direct Q-table with a deep network to estimate Q-values for continuous or large state spaces. This allows for the agent to generalize between similar states and improve learning efficiency in complex or non-stationary environments, such as vehicular networks with varying channel conditions and mobility patterns. DQN also employs mechanisms like experience replay and target networks to stabilize learning. These enhancements, nevertheless, come at the cost of increased memory and processing requirements that may strain the real-time nature of resource-constrained RSUs unless offloaded onto more capable edge servers or supported by hardware accelerators.

The AC algorithm is a hybrid solution that combines the strengths of policy-based and value-based learning. It has two components: the actor, which learns a parameterized policy for action selection directly; and the critic, which criticizes the policy being used with an approximation of the value function. The dual structure supports more stable updates of the policy and more accurate gradient estimation, especially in continuous-action spaces or partially observable settings. AC algorithms tend to achieve faster convergence and improved robustness in dynamic situations, and hence are ideal for those applications where continuous learning and adaptability are of top importance. While slightly more complex than tabular strategies, the computational cost of AC tends to be acceptable, particularly when it is realized using lightweight neural networks and sparsified learning schedules.

Through the use of these four RL agents in a shared simulation framework that emulates RSU-level decision-making over both spectrum allocation and mobility management, the framework provides for a formal side-by-side comparison. This entails a comparison of each agent’s ability to learn effective policies under varying network conditions, responsiveness to hyperparameter settings, convergence rates, and computational burdens. Such an inter-agent inquiry is crucial for determining context-suited RL algorithms that harmonize real-time operation with deployability feasibility in intelligent vehicular networks.

### 4.5. Exploration Strategy

An ϵ-greedy strategy is employed across all agents to ensure a balance between exploration and exploitation. The agent selects a random action with probability ϵ. The exploration rate ϵ decays progressively to promote policy refinement as training progresses.

The RSU agent interacts continuously with a simulated vehicular environment, observes state transitions, selects actions, receives reward feedback, and updates its policy. This learning loop enables the RSU to autonomously optimize spectrum usage, adhere to priority constraints, and dynamically adapt to varying vehicular traffic profiles without requiring explicit traffic models or predefined policies.

## 5. Performance Evaluation

To evaluate the effectiveness of the proposed RL-PASM framework in a V-IoT context, extensive simulations are conducted using a centralized spectrum access model managed by an RSU. Four RL agents are evaluated and compared under varying network load conditions. The objective was to assess the convergence, efficiency, fairness, and scalability of learned policies across multiple performance metrics. The simulation environment models a discrete-time decision process in which three traffic classes, HP, LP, and BE, contend for access to a limited number of wireless channels. At each time step, one of these devices requests access, and the RSU selects an action from a five-action set: deny, grant, preempt, coexist, and hand-off.

Each simulation episode consists of 100 time steps, and agents are trained over 5000 episodes. The learning rate (α) is set to 0.1, the discount factor (γ) is 0.9, and the ϵ-greedy exploration policy starts with ϵ=1.0 and decays gradually to a minimum of ϵ=0.05. For DQN and Actor–Critic agents, the Q-function and policy networks are modeled using a two-layer feedforward neural network. Each hidden layer contains 128 units with ReLU activation, followed by a final output layer sized according to the number of actions. The optimizer used is Adam with a learning rate of $10^{-3}$ and a batch size of 64. A replay buffer of size 5000 is used to store experiences and stabilize training.

The following key performance indicators (KPIs) are recorded for each scheme:

Throughput: Defined as the number of successful transmissions per episode, normalized and scaled according to 5G vehicular network data rates. We assume each successful transmission corresponds to 10 Mbps; hence, total throughput per episode is computed as

(4) $${\mathrm{Throughput}}_{\mathrm{Mbps}}=\frac{\mathrm{Total}\;\mathrm{Successful}\;\mathrm{Grants}}{\mathrm{Steps}\;\mathrm{per}\;\mathrm{Episode}}\times 10$$

Delay: Computed as the average waiting time due to coexistence or hand-off actions. We scale the per-action delay cost assuming one time step ≈ 10 ms, making delay

(5) $${\mathrm{Delay}}_{\mathrm{ms}}=\mathrm{Avg}\;\mathrm{Delay}\;\mathrm{per}\;\mathrm{Episode}\times 10$$

Jain’s Fairness Index: Measures the fairness of spectrum allocation across traffic classes. For *n* classes with throughput $x_{i}$, it is given by

(6) $$F=\frac{{\left(\sum_{i=1}^{n}x_{i}\right)}^{2}}{n\;\cdot \;\sum_{i=1}^{n}x_{i}^{2}}$$

Energy efficiency: Calculated as the ratio of successful transmissions to energy cost, where each action incurs a pre-defined energy weight.

Blocking probability: Fraction of denied access requests over total access attempts.

Interruption probability: The proportion of preemption events caused by higher-priority devices.

Convergence episode: The episode number at which the moving average of rewards stabilizes at 95% of its final value.

Training time: The total time taken to train each agent over all episodes.

Performance was assessed using moving averages, cumulative distribution functions (CDFs), and bar plots. Across all load scenarios, the DQN and Actor-Critic agents demonstrated superior throughput and fairness, while Q-Learning variants offered faster convergence. Blocking and interruption probabilities were minimized under all RL-based schemes, confirming the effectiveness of learned policies.

The RL-PASM framework dynamically adapts to network congestion and traffic heterogeneity, offering a robust, intelligent solution for future 5G and 6G vehicular communication systems.

## 6. Results and Discussion

The framework is evaluated by simulating the spectrum access in a realistic 5G V-IoT environment, where a centralized RSU dynamically manages spectrum access under varying traffic loads and priority-based service requirements. The evaluation spans several key performance dimensions, including learning convergence, spectrum utilization efficiency, service latency, energy optimization, fairness across traffic classes, communication reliability, and training cost.

**Figure 3:**
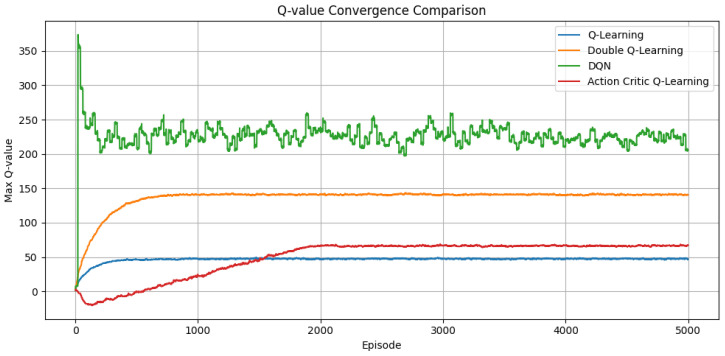
Maximum Q-value trend across episodes for each of the four evaluated RL algorithms.

Figure 3 depicts the evolution of Q-values throughout training. DQN achieves the highest and maintains a stable Q-value, benefiting from non-linear function approximation and experience replay mechanisms. It converges the fastest, while DQL displays a smoother Q-value stabilization than standard QL, reinforcing its ability to avoid overestimated returns and adapt more reliably to fluctuating network states. The AC algorithm shows slightly delayed Q-value stabilization, but ultimately reaches a consistent value regime. This behavior is characteristic of its dual-network architecture, where the actor’s exploration and the critic’s evaluation gradually co-adapt to yield effective policy learning.

### 6.1. Throughput

**Figure 4:**
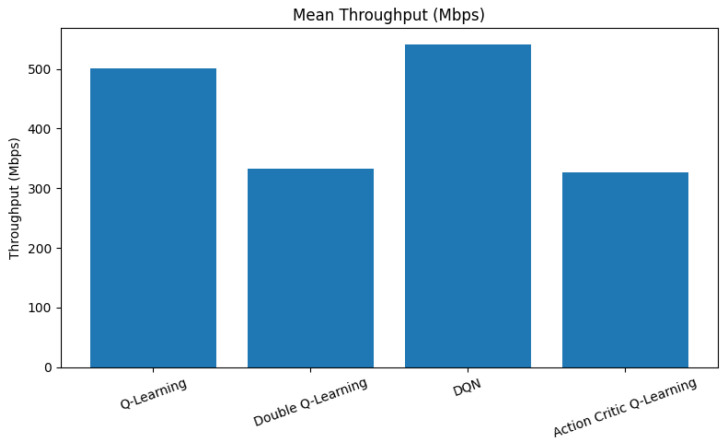
Mean throughput comparison across RL agents.

Throughput is a fundamental metric that reflects how efficiently an RL agent utilizes available spectrum resources to maximize data transmission rates. Figure 4 presents a comparison of average throughput across the evaluated reinforcement learning agents.

As observed in Figure 4, the DQN agent achieves the highest average throughput, surpassing 540 Mbps. This superior performance is attributed to its deep neural architecture, which enables it to learn complex state–action correlations and effectively exploit high-throughput channel states. QL performs moderately well, achieving approximately 500 Mbps. Despite its tabular simplicity, it benefits from aggressive exploration and rapid convergence, allowing for it to learn useful patterns under fixed state-action representations. In contrast, the DQL and AC agents demonstrate lower throughput values, averaging around 330 Mbps. This outcome stems from their more conservative decision-making strategies. DQL, while stable and less prone to overestimation, tends to under-utilize available spectrum during early training phases. Similarly, AC focuses on balancing exploration and exploitation, which can initially limit its throughput performance before convergence is achieved.

### 6.2. Delay Performance

**Figure 5:**
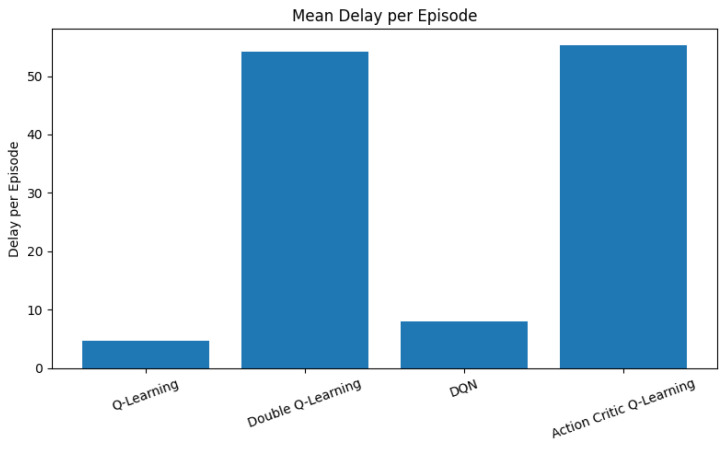
Mean delay per episode across all RL agents in milliseconds (ms).

**Figure 6:**
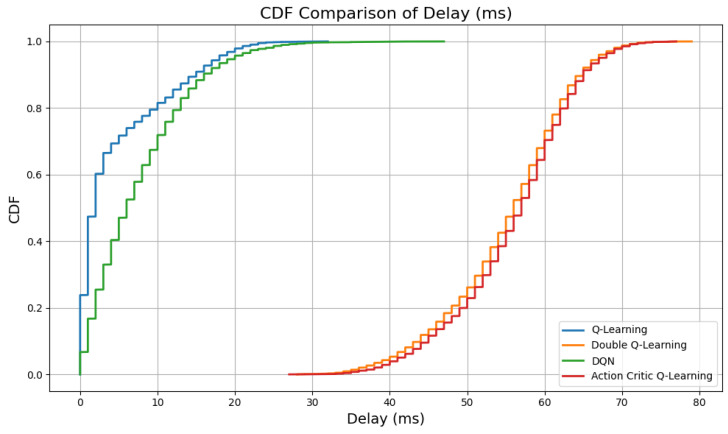
CDF of delay across all agents.

Delay is a critical metric in V-IoT systems, especially for latency-sensitive applications such as collision avoidance and emergency braking. Figure 5 and Figure 6 illustrate the mean delay per episode and the cumulative distribution function (CDF) of delay, respectively, across all RL agents.

As depicted in Figure 5, vanilla QL achieves the lowest average delay, closely followed by DQN. Both agents are effective at minimizing communication latency through frequent execution of grant and pre-empt actions, which quickly assign spectrum resources to high-priority requests. This behavior is particularly beneficial in environments where rapid decision-making is crucial for real-time responsiveness. Conversely, DQL and AC agents exhibit significantly higher average delays. The conservative policy updates in DQL, aimed at reducing overestimation bias, tend to delay the reinforcement of aggressive access decisions. Similarly, the AC framework, which balances two learning objectives through separate actor and critic networks, exhibits slower convergence in prioritizing urgent traffic flows. This results in increased end-to-end latency during the training process.

The distribution illustrated in Figure 6 provides further insight into the delay characteristics. QL and DQN demonstrate steep left-shifted CDF curves, indicating that most episodes incur lower delay values. In contrast, the CDFs for AC and DQL rise more gradually, suggesting that these agents experience a broader range of delay outcomes, with a greater portion of episodes exceeding desirable latency thresholds. However, this increased average delay can be attributed to a smaller number of successful transmissions compared to vanilla QL and DQN.

### 6.3. Energy Efficiency

Energy efficiency is a crucial metric in V-IoT systems, especially for battery-constrained devices and energy-aware applications. It quantifies the ratio of successful transmissions to the energy consumed per episode. Therefore, it reflects the ability of an agent to achieve reliable communication with minimal energy expenditure.

**Figure 7:**
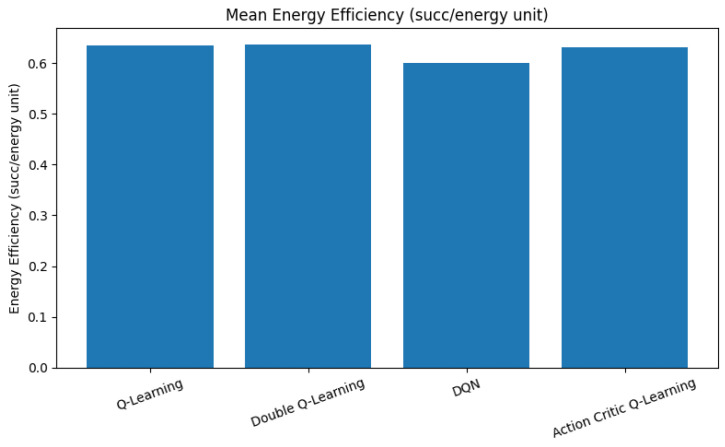
Mean energy efficiency, computed as the successful transmissions per unit energy across all agents.

As illustrated in Figure 7, QL, DQL, and AC agents exhibit comparable average energy efficiency levels, all converging around a value of 0.64. This indicates that these agents balance channel utilization with power-conscious access decisions, often avoiding aggressive actions that may lead to frequent hand-offs or unnecessary spectrum contention. In contrast, the DQN demonstrates a slightly lower average efficiency of approximately 0.60. This reduction stems from its higher throughput objective, which often leads to more aggressive spectrum usage and frequent preemption or coexistence actions. These decisions, while beneficial for maximizing data rates, tend to consume additional energy, resulting in a trade-off between spectral utilization and energy cost.

### 6.4. Fairness

In V-IoT networks, ensuring equitable spectrum access across heterogeneous traffic classes is essential for system stability and long-term service quality. Jain’s Fairness Index is used to quantify how uniformly access opportunities are distributed among these traffic types, with values closer to 1 indicating higher fairness.

**Figure 8:**
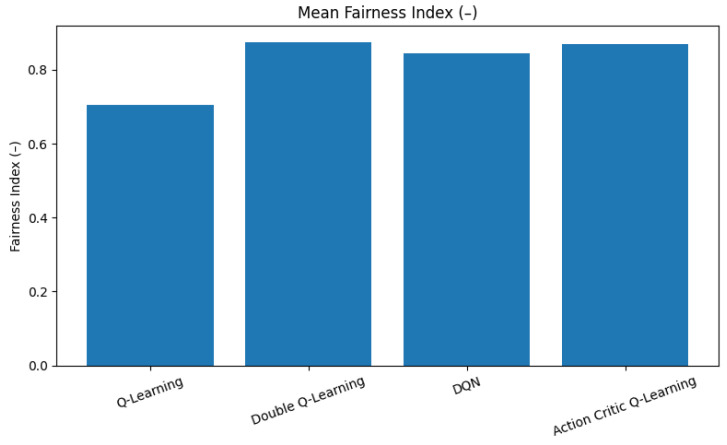
Mean Jain’s fairness index for all RL agents.

As evident in Figure 8, QDL and AC agents achieve the highest average fairness, both exceeding a value of 0.86. This suggests that their policies effectively balance access decisions across all traffic classes, ensuring that no single class dominates the spectrum resources over time. Their underlying learning dynamics, which involve careful reward evaluation and overestimation mitigation in AC and DQL, respectively, contribute to this balanced behavior.

In contrast, QL records the lowest fairness index of approximately 0.70. This reflects its tendency to overly favor high-priority traffic, thereby maximizing immediate rewards and resulting in the underrepresentation of lower-priority traffic. While this can be beneficial for mission-critical tasks, it introduces significant service inequity.

### 6.5. Interruption Probability Analysis

Interruption probability reflects the frequency of service discontinuities caused by spectrum reallocation, particularly due to preemption when a higher-priority request overrides an ongoing transmission. Minimizing such interruptions is crucial in V-IoT networks, particularly for latency-sensitive or safety-critical applications, such as emergency vehicle coordination, collision avoidance, or real-time telemetry.

**Figure 9:**
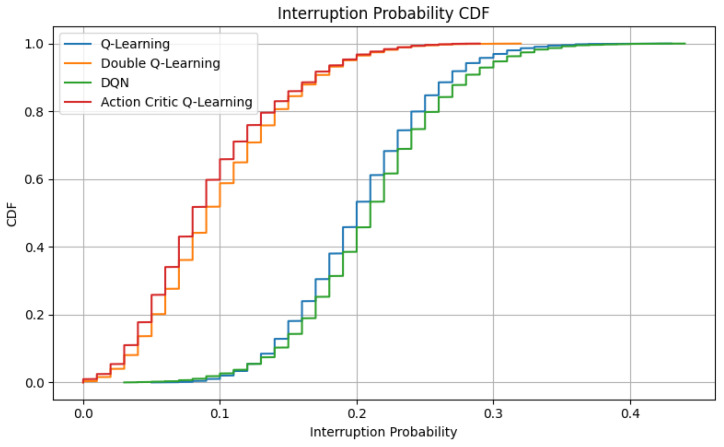
CDF of interruption probability for each RL agent.

Figure 9 presents the CDF of interruption probabilities observed across simulation episodes. It can be seen that the DQL and AC agents exhibit notably low interruption probabilities, with the majority of episodes staying below 10%. This behavior demonstrates their conservative policy strategies, which aim to reduce disruptive access decisions and instead prioritize service continuity and reliability. In contrast, both DQN and QL display broader CDF profiles, with interruption probabilities reaching up to 40% in several episodes. These higher values suggest that these agents, while potentially more aggressive in maximizing throughput or minimizing delay, tend to initiate preemptive actions more frequently, disrupting ongoing communications, particularly for lower-priority devices.

This observation reinforces the strategic differences between learning paradigms. AC and DQL prioritize long-term stability by deferring unnecessary pre-emptions, resulting in smoother traffic flows and fewer session terminations. Conversely, the DQN and QL agents, although efficient in aggressive spectrum utilization, may compromise session reliability under high-load or bursty traffic conditions.

### 6.6. Blocking Probability

Blocking probability represents the likelihood of access request denials due to unavailable channels or overly conservative access decisions. In V-IoT networks, particularly under bursty or high-density traffic conditions, a high blocking rate can significantly degrade QoS, especially for high-priority applications such as safety alerts or vehicular coordination.

**Figure 10:**
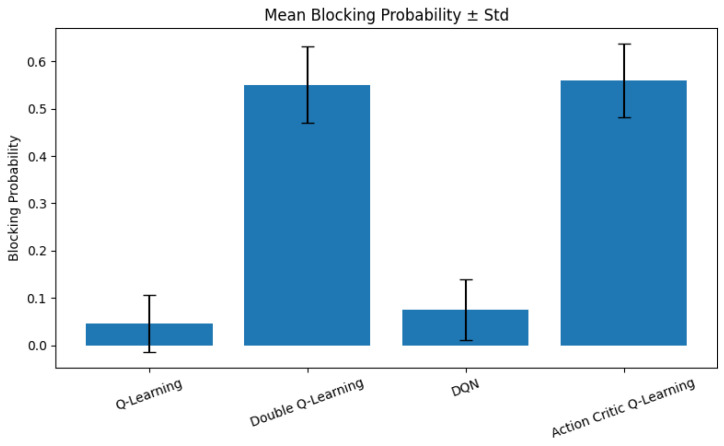
Mean blocking probability with standard deviation across RL agents.

Figure 10 presents the mean blocking probability and standard deviation for all agents. The QL and DQN agents consistently maintain low blocking probabilities, both under 0.1. This is attributed to their proactive access strategies, such as aggressive granting and preemptive channel reassignment, which help ensure higher channel occupancy and reduced denial rates. In contrast, DQL and AC agents exhibit significantly higher blocking probabilities. This behavior stems from their more conservative and cautious policies, which tend to delay or deny access to preserve fairness or prevent interruption. While this restraint improves stability and fairness, it leads to the underutilization of spectrum resources in high-demand scenarios, resulting in frequent access rejections. The standard deviation analysis further corroborates this behavior. QL and DQN not only show lower blocking on average, but also exhibit less variability, suggesting more consistent performance across episodes. Conversely, the wider spread observed for DQL and AC indicates occasional overconservatism, especially during traffic spikes.

These results highlight a key trade-off in RL-driven spectrum management: while aggressive agents, such as DQN and QL, optimize for throughput and access opportunities, they may compromise fairness and stability. On the other hand, conservative agents offer reliability and equity, but may incur significant channel underutilization and service denial, particularly under constrained spectrum conditions. The findings reflect a fundamental trade-off in RL-PASM design. While DQN excels in learning speed and performance, it demands more resources, limiting its applicability in constrained RSU or edge environments. Conversely, tabular methods, although slower in policy optimization, offer rapid deployment capabilities and are well-suited for scalable, low-power vehicular applications.

## 7. Conclusions

This paper introduced RL-PASM, a centralized RL-based PASM framework tailored for V-IoT environments. The framework operates at RSUs and employs four distinct learning agents to make intelligent channel access decisions across heterogeneous traffic classes. By modeling the access process as a discrete-time MDP, the system effectively captures spectrum dynamics and traffic behavior. A carefully designed composite reward function promotes the simultaneous optimization of throughput, fairness, energy efficiency, and class-prioritized equity. Extensive simulation in a 5G-based vehicular setup revealed that DQN excelled in maximizing throughput and spectrum utilization due to its deep representation capabilities. DQL and AC demonstrate superior fairness and stability, while QL and DQN offer notable advantages in delay minimization and energy efficiency, with minimal computational burden. These results highlight the effectiveness of combining RL techniques with priority-aware design in achieving dynamic, equitable, and efficient spectrum access in next-generation vehicular networks.

Building upon this foundation, future work will explore the integration of more advanced and scalable DRL methods, including Proximal Policy Optimization (PPO), Soft Actor–Critic (SAC), and other competitive Actor–Critic variants such as DDPG and TD3, to support continuous and high-dimensional state-action spaces. We also plan to implement federated RL across distributed RSUs to enhance scalability while preserving data privacy. The reward structure will be extended to include mobility-aware factors such as vehicular speed, density variation, and predicted handover frequency, thereby enabling more context-sensitive policy learning. We aim to evaluate RL-PASM using real-world vehicular testbeds or digital twin-based emulation platforms. This will allow for validation under realistic operating conditions that account for wireless channel impairments, protocol delays, hardware limitations, and real-time feedback mechanisms of future 5G and 6G infrastructures.

## Figures and Tables

**Table 1:** Comparison of RL–based spectrum access strategies in V-IoT.

Reference	Underlying Technique	Key Outcomes
[18]	QL	Developed QLOCA, achieving improved throughput and reduced delay for different traffic classes.
[26]	DQN	C Enabled dynamic access mode switching using DQN, improving spectral efficiency.
[20]	DQN	Balanced multiple QoS goals with DQN, improving convergence and access reliability.
[19]	MARL	Reduced collisions and improved fairness using centralized training and decentralized execution.
[21]	Dueling Double DQN	Achieved high capacity and fast learning through advanced DQN architecture.
[27]	QL	Introduced fairness-based reward adjustments to balance rate and resource utility.
[24]	QL	Demonstrated real-time spectrum handoff decisions in hardware implementation.
[25]	QL-based optimization	Improved spectrum policies through PU activity modeling and theoretical analysis.
[11]	DRQN-based Cooperative MARL	Introduced CoMARL-DSA using CTDE for decentralized spectrum access, improving success rate by 14% over DQSA.
[12]	Double DQN	Developed a fairness-aware DSA strategy for D2D-IoT with hybrid access, achieving near-optimal throughput in both uplink and downlink scenarios.
[13]	Adjusted DDPG	Jointly optimized time scheduling and energy management in AB-assisted hybrid underlay CRNs, outperforming traditional overlay/underlay modes.

## Data Availability

Dataset available on request from the authors.
